# Human Endogenous Retroviruses and Their Putative Role in Pathogenesis of Alzheimer’s Disease, Inflammation, and Senescence

**DOI:** 10.3390/biomedicines13010059

**Published:** 2024-12-30

**Authors:** Patrycja Kozubek, Julia Kuźniar, Magdalena Czaja, Hanna Sitka, Urszula Kochman, Jerzy Leszek

**Affiliations:** 1 Student Scientific Group of Psychiatry, Faculty of Medicine, Wroclaw Medical University, 50-369 Wroclaw, Poland; julia.kuzniar@gmail.com (J.K.); magdalena.czaja47@gmail.com (M.C.); hanna.sitka@o2.pl (H.S.); urszulakochman@gmail.com (U.K.); 2 Department of Psychiatry, Faculty of Medicine, Wroclaw Medical University, 50-369 Wroclaw, Poland

**Keywords:** human endogenous retroviruses, Alzheimer’s disease, senescence, neurodegeneration

## Abstract

The human endogenous retroviruses (HERVs) are ancient exogenous retroviruses that were embedded in the germline over 30 million years ago and underwent an endogenization process. They make up roughly 8% of the human genome. HERVs exhibit many physiological and non-physiological functions; for example, they play a role in the development of many diseases. They have been shown to affect carcinogenesis by modifying the expression of host genes through their functions as enhancers and promoters. Additionally, some molecules derived from HERVs may stimulate the immune system. Recently research has been focused on the effect of human endogenous retroviruses on the development of neurodegenerative diseases, including Alzheimer’s disease (AD), which is the most common cause of dementia. AD is also linked to a significant deterioration in quality of life. The article aims to highlight the potential role of HERVs in neurodegenerative diseases such as Alzheimer’s disease and senescence. Moreover, it is estimated that HERVs may be potential targets for diagnosis and therapy of AD.

## 1. Introduction

Endogenous retroviruses (ERVs), belonging to LTR retrotransposons, are remnants of ancient retroviral infections that became integrated into the human genome millions of years ago during the course of evolution. They constitute about 8% of the human genome [1]. The most recently integrated virus is HERV-K (HML-2), which contains nearly complete transcripts in the human genome. HML-2 proviruses have accumulated numerous deletions, insertions, and various recombinations and mutations over time, which results in all HML-2 found in the human genome being defective in terms of replication [2]. However, several proviruses with the classical organization of retroviral genomes have been described. They contain two long terminal repeat (LTR) regions, as well as the *gag*, *pol*, and *env* genes [3]. The *gag* gene encodes structural proteins, *pol* codes reverse transcriptase and integrase, and the *env* gene produces glycosylated proteins, which stand for envelope proteins: a surface and a transmembrane unit [4]. As part of the human genetic material, HERVs play both beneficial roles in various processes and contribute to negative effects. HERVs participate in immune responses, the formation of syncytiotrophoblasts, and cell-fate specification but may also contribute to pathologies, including pathological inflammation, neurodegeneration, and tumorogenesis [5]. Numerous post-insertion mutations in HERV-K (HML-2) have prevented the production of infectious viral particles. However, some have retained limited coding ability. Epigenetic control, which inhibits expression in healthy tissue, is lost during carcinogenesis. Increased expression of HERV-K-derived proteins has been observed in various types of solid tumors. Their involvement in tumor growth, metastatic potential, and treatment resistance is also postulated. HML-2 alterations were detected in samples from patients with lung cancer, breast cancer, melanoma, prostate cancer, hepatocellular cancer, colorectal cancer, leukemia, and lymphoma [6]. HERVs are also associated with the aging process. The activity of the HERV-K (HML-2) provirus at 1q22 is increased in older individuals [7]. Furthermore, there is evidence linking endogenous viruses to neurodegenerative diseases. Some studies indicate an enhanced immune response to HERV-K-encoded proteins in patients with amyotrophic lateral sclerosis. Increased expression of certain HERV-W transcripts and proteins has been demonstrated while examining brain tissue from patients with multiple sclerosis [8]. Finally, analysis of mRNA molecules in brain tissue and cerebrospinal fluid has shown that HERV-K (HML-2) RNA was detectable more frequently in CSF from individuals with Alzheimer’s disease compared with controls [9].

As a narrative review, we surveyed the literature to investigate the relationship between human endogenous retroviruses hidden in our genome and their broad impact on human physiology, as well as their involvement in disease pathogenesis and immune processes.

## 2. The Human Endogenous Retroviruses Characteristics

The human endogenous retroviruses (HERVs) are remnants of retroviral infections in which the viral genome became embedded as a dormant regulatory element within the host germline over 30 million years ago [10,11].

The term ‘endogenous’ states that those viruses no longer produce infectious particles. Endogenization can then be induced by a multitude of factors, for example, mutations, xenotropic restriction, recombination events, and host-antiviral responses [12]. HERVs make up roughly 8% of the human genome [12]. Most recently, the human GRCh37/hg19 genome assembly was analyzed using RetroTector (ReTe) software, leading to the identification and global characterization of >3000 HERV insertions [10]. Although previously it was thought that HERVs are mostly inactive, their presence in our genome has a number of complex functional and evolutionary consequences. HERVs regulate transcriptional networks, provide retroviral motifs that propagate immunity, innervate host physiology, and contribute to the transcriptome. Therefore, they cannot be simply considered “junk” DNA [10,12]. HERVs account for 0.1–0.4% of all translation in distinct tissue-specific profiles [12]. They present only the main genes: *gag*, polymerase (*Pol*), and envelope (*Env*), encircled by two promoter regions, known as Long Terminal Repeats (LTRs). Because of this genomic organization, HERVs are considered simple retroviruses [13].

HERV RNAs can be found in the ribosomes of healthy human tissues [12]. The nomenclature of the human endogenous retroviruses takes into account the primary binding site where reverse transcription begins [13].

HERVs were originally divided into three main classes:

- Class I (retrovirus- and retrovirus-like). This class includes HERV-W and HERV-H,
- Class II (retrovirus-like). This class includes HERV-K elements.
- Class III (Spumaretrovirus-like). This class includes HERV-L and HERV-S.

HERVs exhibit many physiological functions. A very characteristic one includes the participation of *Env* proteins in placenta formation. Syncytin-1 (HERV-W *Env* protein) and Syncytin-2 (HERV-FRD *Env* protein) have conserved fusogenic capacity that enables the formation of the syncytiotrophoblast layer and its fusion with the cytotrophoblast [14].

Physiological expression of HERV-H components has been observed in embryonic stem cells. It is essential for pluripotency [15]. HERVs have been found to be essential for cell identity because of ERV LTR promoter–enhancer activity, which drives non-coding, stem cell-specific transcripts that maintain the undifferentiated state [16].

The abnormal expression of HERVs has been linked to conditions that include malignant, infectious, autoimmune, and neurological diseases [17].

Some examples include the detection of HERV-W Env proteins in the plasma of patients with schizophrenia and bipolar disorders. Expression of HERV-W Env was significantly upregulated in patients suffering from type 1 diabetes and HERV-H/env59 in patients with systemic lupus erythematosus, and it showed a negative correlation with pathogenetic factors of human autoimmune rheumatic diseases. Multiple sclerosis-associated retroviruses (MSRV) might have formed from the recombination of different HERV-W transcripts. MSRV is associated with a worse prognosis and a higher risk of clinical re-exacerbations in patients with multiple sclerosis [15]. Additionally, in the study analyzing autistic patients, the presence of increased HERV-H transcriptional activity was detected in all subjects [10]. As a result of the crossover between HERV-I loci on the Y chromosome, deletion of an azoospermia factor-a region occurs, which leads to male infertility [18]. The connection between the intestinal microbiomes and HERVs is also significant. Intestinal colonization with bifidobacterium causes global modulation of human endogenous retroviruses [10].

The most recently acquired HERV group was HERV-K, with 11 subgroups, including HML-1 to HML-11. HERV-K has been shown to be the most active group of human endogenous retroviruses (especially its subgroup HML-2). Its copies can produce non-infectious virus-like particles (VLPs).

HERV-K is not expressed in normal adult cells, but it has been observed to be activated and expressed in amyotrophic lateral sclerosis, various cancers, and during HIV-1 infection [19]. In the case of HIV infection, for example, the absence of pericentromeric K111 proviruses is connected with improved HIV management even in the absence of antiretroviral therapy [20]. In recent studies, a positive feedback loop between endogenous retrovirus activation and aging has been discovered. This correlation is present in studies involving yeast, fly, and rodent models. Additionally, in human tissues and serum from the older patients, activation of ERVs was observed. There is also a relationship between HERV activation and the onset of age-related diseases (for example, neurodegenerative diseases and rheumatoid arthritis) [1].

Human endogenous retroviruses can induce deleterious effects through oncoprotein-mediated toxicity, recombination, and insertional mutagenesis [21].

HERV-H transcripts have been detected in cell lines of various different cancer types, for example, lung cancer, teratocarcinoma, colorectal cancer, bladder carcinoma, and testicular tumors. Expression of the HERV-H *env* gene was detected in cancer cells of the brain, prostate, kidney, liver, and lung but was not detected in normal tissues [15]. In addition to HERV-H, other HERVs (HERV-K, HERV-H, HERV-R, HERV-W, HERV-F, and HERV-S) have been detected in cancer cells of several tumors. It has been observed that the HERV-K *Env* proteins have a direct effect on carcinogenesis. They also induce uncontrolled cell fusion in tumors, which leads to metastasis and therapy resistance [21].

HERV-K *Env* proteins were expressed in the majority of primary breast tumors, and in patients with hepatocellular carcinoma, HERV-K expression was associated with worse outcomes [15]. HERV-K is also linked to prostate cancer and pancreatic cancer [15]. Many factors can upregulate HERV-K; this include tumor viruses: Epstein-Barr virus, hepatitis B virus, human T-lymphotropic virus 1, and KS-associated herpesvirus [21].

It is important to deepen the knowledge of HERVs in order to use them as one of the target points of therapy [22]. For example, a monoclonal antibody against an HERV-W *Env* protein is under clinical trial as a therapeutic option for MS [18].

**Figure 1:**
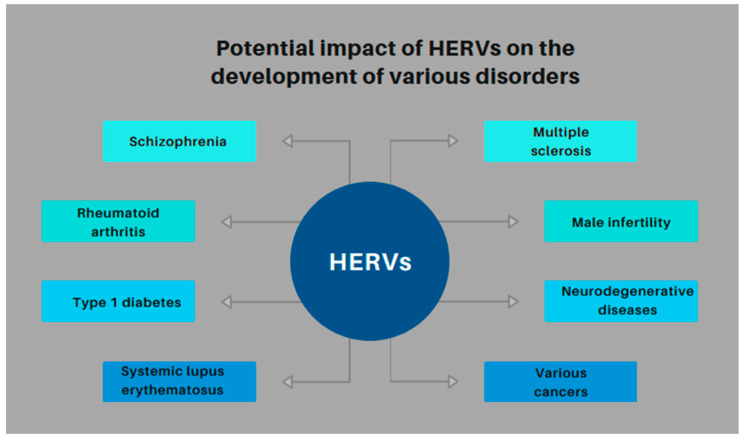
Potential impact of HERVs on the development of various disorders—a summary.

The potential impact of HERVs on the development of various disorders is summarized in Figure 1.

## 3. Mechanism of Action

### 3.1. HERV Impact on the Immune System

HERVs have been an integral part of the human genome for millions of years. During embryonic development, they undergo strong transcription, the effects of which are revealed at multiple levels in the tissues of the adult human body [23]. Sequences associated with HERVs resemble elements of modern retroviruses, including the 5′ and 3′ long terminal repeats (LTR) and the coding genes *env* [24]. However, the long-term presence in the host genome has led to the accumulation of numerous genetic mutations, such as substitutions and deletions, which have ultimately modified their structure [25]. Depending on the immunological environment and the type of cells, molecules derived from HERVs may be tolerated by human defense mechanisms or may stimulate the immune system, which can be significant in the development of various autoimmune diseases. It has been shown that the presence of other immunological stimuli can influence HERV expression. Innate immune mechanisms activated by-products derived from HERVs are the same as those involved in the first line of defense against exogenous retroviruses. HERV expression products have been shown to act as pathogen-associated molecular patterns (PAMPs), triggering cellular receptors responsible for the first line of defense. Additionally, they can provide antigenic epitopes recognized by lymphocytes through molecular mimicry with exogenous viral molecules. These processes stimulate the emergence of specific T and B cells. Consequently, this promotes further inflammatory responses and likely contributes to the development of autoimmune processes and carcinogenesis [23].

Looking more closely at the process of “viral mimicry” that activates the pro-inflammatory pathway, it is worth paying attention to the retinoic acid-inducible gene I (RIG-I)-like receptors (RLRs). The RLR group consists of RIG-I, melanoma differentiation-associated gene 5 (MDA5), and a truncated receptor, the laboratory of genetics and physiology 2 (LGP2). The first two contain 2CARD—a tandem caspase recruitment domain, which downstream signaling through mitochondrial antiviral-signaling protein (MAVS), an adapter protein for RLR signal transduction. The activation of MAVS leads to the translocation of transcription factors IRF7, IRF3, and NF-κB. In response, the RIG-I/MDA5/MAVS pathway induces the expression of genes that contain interferon-stimulated response elements (ISRE) and NF-κB binding sites. As a result, there is an upregulation of type I interferon (IFN-I) and other inflammatory cytokines, contributing to the development of inflammation [26].

LINEs are non-LTR retrotransposons in the genome. The mobilization of LINEs and HERVs is likely associated with disturbances in alternative splicing and epigenetic changes, leading to alterations in gene expression, which is why their expression is usually suppressed through processes such as histone modification. However, some of them are active, such as syncytin-1, which is involved in the formation of syncytia. Syncytin-2 additionally plays a role in immune tolerance, while the expression of HERV-H is involved in maintaining the identity of human stem cells [27].

ERV sequences have significant consequences for shaping the host phenotype due to their ability to integrate with the genome, which can impact physiological functions and play a role in disease pathogenesis. It is believed that syncytins, acquired millions of years ago, such as syncytin-1, which is an envelope protein of ERV encoded by a HERV-W provirus *env*, play a crucial role in the morphogenesis of the placenta. It is postulated that syncytins may have immunomodulatory effects, influencing immunosuppression and inhibiting Th1 cytokine production. Overall, the fetus in the mother’s womb experiences immune tolerance during pregnancy. Furthermore, HERVs serve as key regulatory factors for pluripotent embryonic stem cells [24].

Most HERVs are subject to epigenetic repression, and their ability to encode proteins is limited. However, some HERVs are transcribed due to strong promoter activity from the flanking long terminal repeats (LTR). They retain their open reading frames (ORFs) in the human genome, giving them the potential for transcription and translation. Intermediate replication products may influence the induction of interferon (IFN) as a form of ’viral mimicry.’ In this way, they act like viral infections, sensitizing cancer cells to immune recognition. The proteins produced from these processes can form antigens recognizable by T cells and B cells. Cytotoxic T-cells respond against HERV-derived epitopes in such cancer types as colorectal cancer, breast cancer, renal clear cell carcinoma, melanoma, and seminoma [28].

### 3.2. HERV Activation and the Role of HERV in Cancer Development

In most tissues, DNA methylase-1 catalyzes the transcriptional silencing of HERVs through CpG methylation. HERVs can likely be activated by numerous stimuli, such as hormones, cytokines, or exogenous microorganisms. They are also subject to epigenetic regulation, indicating that certain viral infections, such as those caused by influenza virus, retroviruses (HTLV-1, HIV), and herpesviruses (EBV, HSV-1), may activate HERVs. Potentially oncogenic properties may also occur at the genomic level, including non-allelic recombination of HERV sequences, leading to rearrangements within chromosomes [24]. The intact provirus contains four open reading frames (ORFs). *Gag* encodes core proteins, pro encodes protease, *pol* encodes reverse transcriptase and integrase, while *env* encodes envelope proteins [27]. HERV long terminal repeats (LTR), containing proviral sequences such as *gag*, *pro*, *pol*, and *env* genes, can serve as alternative promoters for the expression of cellular genes, contributing to tumorigenesis. The effects of this have been observed in prostate cancer, Hodgkin’s lymphoma, and diffuse large B-cell lymphoma [24]. They participate in carcinogenesis by modifying the expression of host genes through their functions as promoters and enhancers [29]. In Hodgkin’s lymphoma, transcription of CSF1R is initiated at the LTR element of the MaLR family THE1B, rather than from its own promotor. According to findings, HERV-K proviruses are active as promoters in cancerous epithelial cells of the human breast. Mature cells can be reprogrammed, giving them the potential for self-renewal, which promotes the development and persistence of the tumor process. The influence of HERV has also been observed in testicular cancer. A protein called Testicular Zinc Finger Protein (TZFP), likely a transcriptional regulator during spermatogenesis, binds to the helper protein HERV-K (HML-2) and leads to the release of gene expression for AR and MYC in testicular cancer. The presence of HERV has also been associated with epithelial ovarian cancer (EOC). Compared to healthy tissue, increased levels of HERV-K *Env* have been observed in ovarian cancer tissue. Furthermore, hypomethylation of HERV-K was linked to platinum resistance, leading to decreased progression-free survival and overall survival in clear cell ovarian carcinoma (OCCC). *Env* proteins also appear to play a role in the invasiveness of endometrial cancer. The expression of syncytins was increasing with cancer progression. Syncytin-1 induces cell adhesion and proliferation, invasion, and migration in endometrial cancer. Abnormal HERV expression has also been noted in malignant tumors of the urinary tract. Overexpression of syncytin-1 is more commonly found in tissues of urothelial bladder cancer. Additionally, tumors with such expression exhibit greater aggressiveness. Detection of mutations in the 3′ LTR of HERV-W may serve as a prognostic indicator of more aggressive development [29]. A noticeable increase in the expression of np9, an HERV-K-associated gene, as well as an increase in the RNA expression level of HERV-K *gag*, has been observed in colorectal cancer tissues [30]. The involvement of HERV has also been discovered in hepatocellular carcinoma (HCC). Syncytin-1 leads to the activation of the MEK/ERK pathway, promoting hepatocarcinogenesis. Additionally, it inhibits doxorubicin-induced apoptosis through the MEK/ERK cascade, indicating that syncytin-1 not only drives the progression of HCC but also contributes to the resistance to doxorubicin [31]. The *env* mRNA of HERV R, P, K, and H was also compared in the blood of a healthy control group and in the blood of patients with lung cancer, revealing increased expression of *env* in cancer patients [32]. The role of HERV has also been observed in tumorigenesis in melanoma, where viral envelope proteins induce cell–cell fusion or epithelial-to-mesenchymal transition (EMT) [33].

**Figure 2:**
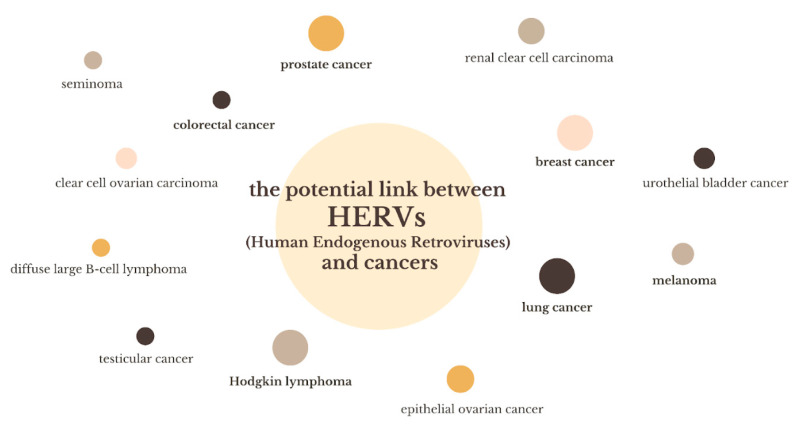
Potential link between HERVs and cancers.

The potential link between HERVs and cancers is presented in Figure 2.

### 3.3. HERV and Its Role in the Inflammatory Response

The expression of HERV genes may be associated with the activation of inflammatory and immunological pathways in the immune system through their influence on the levels of DNA transcription factors, such as cAMP response element-binding protein (CREB) and nuclear factor kappa-light-chain-enhancer of activated B cells (NF-κB) [34]. The role of oxidative stress in the development of inflammatory processes is undeniable. It is caused by an imbalance between oxidants and antioxidants, disrupting the homeostasis of redox processes. Reactive oxygen species (ROS) and reactive nitrogen species (RNS) play a role in signaling as well as damaging biological systems. There is evidence suggesting that the integration of endogenous viruses into the human genome could lead to mutations in the gene that produces ascorbate, an antioxidant. There is a relation between oxidative stress and HERV expression. Abnormal expression of HERVs is associated with inflammatory and neurodegenerative processes, as well as neurological diseases such as multiple sclerosis and amyotrophic lateral sclerosis. Oxidative stress in specific areas of the central nervous system has been linked to the upregulation of transcription factors for HERV-W/HERV-H [13]. There are data indicating that regardless of the primary trigger of the disease, patients with multiple sclerosis exhibit increased presence and expression of the immunopathogenic HERV-W/MSRV. In animal models reproducing the disease, it has been shown that the HERV-W/MSRV *env* protein alone promotes allergic encephalitis and myelitis in mice [35]. The *ENV* protein of the MSRV virus, which is HERV-W associated with multiple sclerosis, acts as a strong agonist of toll-like receptor 4 (TLR-4), a pattern recognition receptor in innate immunity, triggering an inflammatory response. It increases the release of cytokines such as TNF-α and interleukin-6 from human peripheral blood mononuclear cells [36]. Syncytin-1, the same protein that is involved in placenta development, can induce inflammation in astrocytes and microglial cells when expressed abnormally [37]. Inflammation, carcinogenesis, embryogenesis, and the research on the functioning of nucleic acids and proteins derived from ERVs demonstrate the impact of HERVs on human biology. Some authors suggest that modulation of ERVs in preclinical studies on autoimmune diseases and cancer has been approached using epigenetic therapies or reverse transcriptase inhibitors. The topic of generating viral mimicry and inducing an interferon response, as well as studies on ERV dysregulation and molecular pathways, seems to have potential in the therapy of related diseases [38].

## 4. Role of HERVs in AD and Senescence

Alzheimer’s disease (AD) is the most common cause of dementia around the world because of the aging population. It is estimated that, by 2050, the prevalence of dementia will double in Europe and triple worldwide. The disease is becoming one of the most expensive, multifactorial, incurable, and burdening health problems of this century [39].

The pathogenesis of AD is multifactorial—it is characterized by neuritic plaques as a result of amyloid-beta peptide’s (Aβ) accumulation in the brain and neurofibrillary tangles composed of hyperphosphorylated tau (especially in the medial temporal lobe and neocortical structures) and the gradual loss of synapses.

It results in progressive loss of cognitive functions, changes in mood, anxiety, depressive symptoms, and apathy. Then there is a progression to later-stage symptoms, such as loss of memory, impaired judgment, disorientation, confusion, aggression, agitation, delusions, and hallucinations [40]. However, it remains unclear what primarily triggers and causes the progression of the disease. Despite much research, targeted therapies are unsuccessful in reversing the disease or halting its development [41]. The pathogenesis of AD has long been under intense investigation. Age, sex, genetics, and environment are all known to play roles in the development of the disease, with neuroinflammation considered to be a major component. For this reason, researchers have begun to consider the involvement of HERVs in the pathogenesis of Alzheimer’s disease [42].

### 4.1. HERVs, Alzheimer’s Disease, and Senescence

HERVs come from the Retroviridae family, which are retroviruses that have entered the human genome by infection of an ancestral germline [41]. The majority of HERVs do not code functional proteins. However, research showed that dormant HERVs may be “awakened” and linked to the aging processes in young cells as a result of the production of retrovirus-like particles by senescent cells [41].

#### 4.1.1. HERVs Activation

The majority of endogenous retroviruses are thought to be in a dormant state and suppressed by molecular mechanisms. However, epigenetic repression can break down, leading to a reactivation, initiating disease onsets and progression. Factors such as infections, nutrition, stress, hormones, and drugs (aspirin, caffeine, valproic acid) are related to play roles in the process of epigenetic unlocking. Studies unveiled that inflammation has a primary role in stimulating ERV induction, as revealed by correlations between the expression of HERVs with injuries such as burns, trauma, hypoxia, and septic shock. Whereas none of these injuries are primarily associated with risk factors for developing neurological disorders, all of them are linked to the inflammatory reaction and upregulation of different ERVs [43,44].

Endogenous retroviral elements need to be epigenetically unlocked before they can be activated. Processes that are relevant for controlling ERV expression are as follows: localization of proviruses in the heterochromatin, blocking long terminal repeat (LTR) access, CpG methylation, and histone deacetylation. There are several possible processes that are responsible for the re-awakening of retroviral elements from a silenced state via histone acetylation and methylation. For instance, ultraviolet radiation exposure is associated with alterations in DNA methylation, DNA methyltransferase activities, and histone acetylation, which activate the HERV-K *pol* gene and expression of *Env* protein [44]. Moreover, valproic acid, vorinostat, decitabine, and hydroquinone may be responsible for the upregulation of human ERV elements by altered DNA methylation. The next epigenetic change responsible for HERV reactivation is the loss of the histone variant H3.3, which leads to a reduction of suppressing H3K9me3 marks at ERV elements. This in turn would open up binding sites for the interferon regulatory factor family of transcription factors. Moreover, viral infections could be one of the environmental factors linking altered ERV activity to neurological and psychiatric disorders. Exposure of B cells to EBV was found to cause a genome-wide activation of LTR sequences, which further coincided with local DNA hypomethylation [44].

HERV-H, HERV-K, HERV-L, and HERV-W are transcriptionally active in the brains of AD patients. This activation is mediated via heterochromatin relaxation and loss of epigenetic host control over HERVs. Upregulation of ERV-K in a streptozotocin murine model of sporadic AD was associated with cognitive impairments in contextual fear memory and spatial learning [43]. Additionally, in patients with MS, the expression level of HERV-W in the brain correlates positively with the severity of disability and disease progression [43].

#### 4.1.2. The Role of Extracellular Vesicles (EVs)

The role of extracellular vesicles (EVs), for instance, retroviral-like particles (RVLPs), is a recent topic of research. Studies have found connections between the increased amount of EVs and [45].

Extracellular vesicles are considered to be bilayer lipid membrane-enclosed particles that may be secreted by almost any cell type, such as neurons.

Human endogenous retrovirus transcripts and proteins may be transported in EVs, including retroviral-like particles (RVLPs), and act as paracrine signaling for continued pluripotency and proliferation of the cells [45].

EVs with HERV-K proteins inside can operate by paracrine signaling. It results in inflammation and destruction in other tissues, especially in young cells. Moreover, HERV-K RNA or cDNA packaged into extracellular vesicles have the opportunity to avoid recognition of the immune system [45].

It is estimated that ERV activation is connected with hippocampus-related learning disturbances in mice (mediated by the cytosolic RNA sensor MAVS). Therefore, HERVs may be a molecular target for strategies to treat dementia and neuropsychiatric disorders [46].

#### 4.1.3. The Role of TAU Protein and Neuroinflammation

It is estimated that the tau protein induces chromatin relaxation, DNA breaks, and disturbed transcription of genes, which may activate TEs (transposable elements) and result in neurodegeneration. Research of transgenic Drosophila models showed that tau pathology activates retrotransposons by heterochromatin relaxation. Neurofibrillary tangles are linked to increased HERV-Fc1 transcripts, suggesting tau pathology may activate HERV transcription. In the mouse model, TE-derived proteins and TE DNA were elevated in the brain compared to a control group. It suggests that there is a pathological relationship between tauopathy and TE transposition [41].

Studies suggest that TE activation in AD is a consequence of pathogenic forms of tau. Human analyses determined that differential retrotransposon expression correlates with the burden of tau, but not Aβ, pathology in AD. New studies suggest that the feedback between ERV expression and the intercellular spread of TDP-43 may be broadly applicable to other aggregation-prone proteins involved in neurodegenerative disorders, including tau [47].

In AD brains, microglia may convert to a neuroinflammatory condition characterized by the increased production of inflammatory substances, elimination of protein aggregates, and pruning of synapses. These changes activate TE expression; however, TEs could also start neuroinflammation. In mice, disturbance of the co-repressor protein Trim28 resulted in H3K9me3-dependent ERV activation in proliferating progenitor cells. Moreover, neuronal ERV expression was related to the activation of microglia and ERV protein aggregate-like structures [42].

Recent research has shown that pTau promotes neuronal cell cycle reentry. It results in aneuploidy, hyperploidy, and mosaicism typical in neurodegenerative diseases such as AD. Moreover, Tau impairs the genome, activates transposable elements, and causes neuroinflammation. pTau was also responsible for altering αV/β1 integrin, transforming trophic into neurotoxic astrocytes [48].

#### 4.1.4. The Role of HERV-K

Human Endogenous Retrovirus K (HERV-K) has multiple open reading frames for viral proteins—*Gag*, Reverse Transcriptase (*RT*), Envelope (*Env*), and Protease (*Pro*) [45]. HERV-K RNA had a larger frequency in patients with AD than in healthy subjects in the cerebrospinal fluid. These RNA-elevated levels caused neurodegeneration due to the activation of human toll-like receptor TLR8 (HERV-K transcripts containing a motif 5′-GUUGUGU-3′ contribute to neuronal death and microglial accumulation [43] and murine TLR7 in microglia and macrophages, with the release of TNF-α and induction of NF-κB. HERV-K RNA also affected neurons, promoting injury and apoptosis [49]. As has been observed in other studies, HERV-K reactivation, or “resurrection”, is shown to be caused by reduced DNA methylation of its promoters as well as open chromatin in aged cells [45].

#### 4.1.5. The Role of gencDNAs and Reverse Transcriptase

Studies show that neurons from the AD brain cortex have increased DNA content by 8% relative to neurons from age-matched control brains (especially increased copy numbers of the APP gene). Moreover, they revealed neuronal insertions of DNA that come from APP mRNA—gencDNAs. GencDNAs are characterized by a shortage of introns, point mutations, and exon deletions that can be transcribed and translated. It results in the production of abnormal proteins. The mRNAs go through many cycles of retro-insertion that result in errors that link to amyloid aggregation [50]. Creation of gencDNA requires the following: expression of the gene, reverse transcription, and breaks in the genomic DNA strands. However, humans lack intrinsic reverse transcriptase; therefore, the enzyme might be provided by a human endogenous retrovirus or a retrotransposon, which are activated under conditions such as herpetic infections or neuroinflammation. Some studies suggest that patients receiving reverse transcriptase inhibitors may show lower AD risk, but the evidence for this correlation requires further research [50].

Identification of locus-specific TEs linked to AD pathogenesis leads to searching for new therapeutic methods and new reverse transcriptase inhibitors that may reduce neuroinflammation [42].

**Figure 3:**
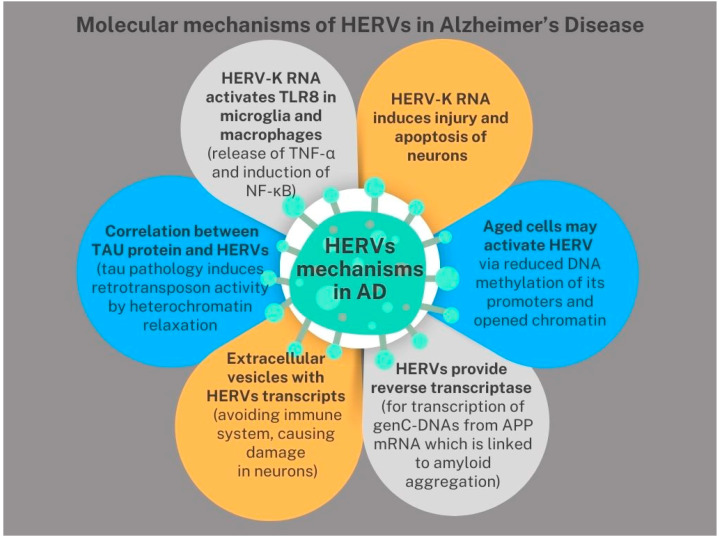
The molecular mechanisms of HERVs in AD.

The summary of the molecular mechanisms of HERVs in AD is presented in Figure 3.

### 4.2. Neuroinfection and the Role of HERVs

Not only HERVs may cause neurodegeneration, but also regular exogenous human retroviruses such as HIV may be a reason for neurological damage [35]. Neuronfection has the potential to activate inflammation and stimulate HERVs in the brain. Permanent inflammatory processes lead to the neurodegeneration observed in AD [41,51]. Chronic HERV activation may cause progressive cognitive impairment and dementia in susceptible people [51].

Factors that are linked to the innate immune response, such as NF-κB, IRF-1, IRF-3, and more, present the potential to activate the endogenous retrovirus LTR, suggesting that immune stimulants (viruses, bacteria, etc.) contribute to premature aging-related phenotypes [45]. Repetitive endogenous re-infections by Herpes family viruses and chronic exposure to environmental factors might be responsible for inducing retrotransposon mutations [51].

### 4.3. Inflammatory Response and HERVs

There are two possible mechanisms that are linked to inflammation caused by HERVs. First, HERV-derived elements cause nonspecific innate immune processes. Second, HERV RNA or proteins may stimulate selective signaling mechanisms [52]. The HERV *Env* protein (as a superantigen) promotes inflammation, cytotoxicity, and apoptosis. It causes stimulation of T lymphocytes, which leads to massive cytokine release. HERV-W *Env* promotes dendritic cell maturation through the Th1-like immune response and stimulates CD14/toll-like receptor 4 (TLR4) [52,53].

Moreover, RNA in the HERV-K *env* gene domain binds to TLR7 in mouse neurons and TLR8 in human neurons. The HERV-W *Env* protein binds to TLR4 and CD14 and activates the production of cytokines such as IL-1β, IL-6, and TNF-α [52].

These findings suggest that neurodegeneration in Alzheimer’s disease may be associated with HERVs.

Potential drugs that may be taken under consideration in the therapy of Alzheimer’s Disease are Sodium Glucose Cotransporter 2 Inhibitors (SGLT2i). Diabetes is characterized by similar pathophysiological mechanisms with dementia, for instance: systemic inflammation, oxidative stress, insulin resistance, and advanced glycation end-product formation. SGLT receptors are localized in brain parts that are involved in the learning process and food intake regulation. The antioxidant action of SGLT2i therapies may synergistically affect pathways involving HERVs in AD’s pathophysiology because of the similarities in causing oxidative stress and neuroinflammation. SGLT2i are linked to anti-apoptotic, anti-inflammatory, and antioxidant effects. Moreover, these drugs reduce the hyperphosphorylated tau levels and amyloid β accumulation and enhance brain insulin signaling. Their effects may be used in the inhibition of HERV activation and prevention of neuronal apoptosis caused by HERV proteins and RNA.

However, there is insufficient scientific evidence to recommend their use in patients diagnosed with AD. There is a high need for future research on this topic [54].

### 4.4. Multiple Sclerosis, Amyotrophic Lateral Sclerosis, and HERVs

In addition to Alzheimer’s disease, links have also been found between HERVs and other neurological impairments. There is a significant correlation between HERV-W expression and multiple sclerosis (MS). Research also showed links between HERV-K and amyotrophic lateral sclerosis [35]. Lymphocytes from patients with MS produce reverse transcriptase (RT)-positive retrovirus-like particles containing HERV-H and/or HERV-W/MSRV sequences [55].

To sum up, the role of HERVs in AD pathogenesis is unclear, and more research is needed.

## 5. Conclusions

Human endogenous retroviruses are involved in the development of various diseases. Those include cancer, autoimmune, and neurological disorders. As an example, in the case of cancers, ERs are a source of enhancers that present cancer-specific activity and can shape cancer-specific gene regulation, including oncogene expression. Neurodegenerative diseases with more established links to abnormal HERV activity include multiple sclerosis and amyotrophic lateral sclerosis. In this work, we focused on the relationship between HERVs and the pathogenesis of Alzheimer’s disease. We also believe that in the future, HERVs could be used as therapeutic points for AD and other neurodegenerative disorders, for example, by reducing virus-associated neuroinflammation and many more. Recently a monoclonal antibody against an HERV-W *Env* protein has been under clinical trial as a therapeutic option for MS. Although the expression of human endogenous retroviruses is strictly regulated in our genome, many factors (endogenous and exogenous) may activate HERV activation. The impact of human endogenous retroviruses on the development of AD and other neurodegenerative diseases is multidirectional.

Dysregulation of human endogenous retroviruses is related to the development of neurodegenerative mechanisms. Dysregulation of HERVs can result from inflammation of small areas of the brain initially caused by brain trauma or viral infections. Chronic HERV activation in genetically susceptible individuals can cause progressive neurodegeneration, which then combines with cognitive impairment and dementia.

Additionally, HERV-K proteins included in EVs, via paracrine signaling, may result in inflammation and damage in young or non-senescent cells of other tissues. HERV-K expression is elevated in the brain transcriptomes of patients with AD compared to a control group and its transcripts in CSF are also significantly elevated in AD. HERV-K is the most expressed and differentially upregulated TE in Alzheimer’s disease.

It is important to conduct further studies and increase knowledge of the impact of HERVs on the development of neurodegenerative diseases, including Alzheimer’s disease, in order for HERVs to become one of the target points for therapy.

## Data Availability

Data sharing is not applicable, as no datasets were generated or analyzed during the current study.
